# An ApoA-I Mimic Peptide of 4F Promotes SDF-1α Expression in Endothelial Cells Through PI3K/Akt/ERK/HIF-1α Signaling Pathway

**DOI:** 10.3389/fphar.2021.760908

**Published:** 2022-01-17

**Authors:** Kaixuan Lv, Lingyu Kong, Mei Yang, Linlin Zhang, Shangmin Chu, Lichun Zhang, Jielun Yu, Guoshen Zhong, Yanhua Shi, Xia Wang, Nana Yang

**Affiliations:** ^1^ School of Bioscience and Technology, Weifang Medical University, Weifang, China; ^2^ School of Rehabilitation Medicine, Weifang Medical University, Weifang, China; ^3^ Medical Laboratory Animal Center, Weifang Medical University, Weifang, China; ^4^ Weifang Key Laboratory of Animal Model Research on Cardiovascular and Cerebrovascular Diseases, Weifang, China; ^5^ School of Public Health and Management, Weifang Medical University, Weifang, China

**Keywords:** apolipoprotein A-I, human umbilical vein endothelial cells, stromal-cell-derived factor-1α, hypoxia-inducible transcription factor-1 alpha, 4f

## Abstract

Atherosclerosis (AS) seriously impairs the health of human beings and is manifested initially as endothelial cells (ECs) impairment and dysfunction in vascular intima, which can be alleviated through mobilization of endothelial progenitor cells (EPCs) induced by stromal-cell-derived factor-1α (SDF-1α). A strong inverse correlation between HDL and AS has been proposed. The aim of the present work is to investigate whether 4F, an apolipoprotein A-I (apoA-I, major component protein of HDL) mimic peptide, can upregulate SDF-1α in mice and human umbilical vein endothelial cells (HUVECs) and the underlying mechanism. The protein levels of SDF-1α were measured by ELISA assay. Protein levels of HIF-1α, phosphorylated Akt (p-Akt), and phosphorylated ERK (p-ERK) were evaluated by Western blotting analysis. The results show that L-4F significantly upregulates protein levels of HIF-1α, Akt, and ERK, which can be inhibited by the PI3K inhibitor, LY294002, or ERK inhibitor, PD98059, respectively. Particularly, LY294002 can downregulate the levels of p-ERK, while PD98059 cannot suppress that of p-Akt. D-4F can upregulate the levels of HIF, p-Akt, and p-ERK in the abdominal aorta and inferior vena cava from mice. These results suggest that 4F promotes SDF-1α expression in ECs through PI3K/Akt/ERK/HIF-1α signaling pathway.

## Introduction

As the leading cause of cardiovascular and cerebrovascular diseases, atherosclerosis (AS) seriously impairs the health of human beings (Torres et al., 2015). It has been suggested that endothelial cells (ECs) impairment and dysfunction in vascular intima act as the initial event of AS onset (Schwartz et al., 2010). Therefore, the restoration of impaired ECs contributes to the suppression of AS development. Endothelial progenitor cells (EPCs) can differentiate into ECs as the precursor cells of the latter. EPCs are involved in vascular integrity, function, and repair, along with angiogenesis (Asahara et al., 2011). Many studies have shown that EPCs can migrate to the damaged site in endothelium and mediate its regeneration, promote the neovascularization in ischemic lesions, and thus alleviate the progression of AS (Hill et al., 2003; Naito et al., 2012; Xu et al., 2014). Stromal-cell-derived factor-1α (SDF-1α), a well-known chemokine expressed in multiple tissues and cells such as ECs, has been demonstrated to be related to cardiac protection (Zaruba and Franz, 2010). Upregulated SDF-1α in ischemic tissues has been shown to be capable of mediating the mobilization of BM-derived EPCs into peripheral blood and their homing to the damaged site and then promoting the neovascularization of ischemic tissues (Yin et al., 2010; Rath et al., 2016). It has been observed that selectively expressed SDF-1α in impaired tissues is implicated in adult stem cell recruitment and tissue regeneration, suggesting its vital role in recruitment of stem and progenitor cells (Askari et al., 2003; Yamaguchi et al., 2003).

A strong inverse correlation between high-density lipoprotein (HDL) and AS has been proposed by many epidemiological studies even after the adjustment of many risk factors, although the correlation is not a causal one (Toth et al., 2013; Karathanasis et al., 2017). Classically, HDL works mainly through reversing cholesterol transport (RCT) and delivering cholesterol from peripheral tissues into the liver and steroidogenic organs (Connelly and Williams, 2004). HDL exerts preventive effects on AS mainly through its major structural protein of apolipoprotein A-I (apoA-I) (Tardif et al., 2007).

It has been known that hypoxia-inducible transcription factor-1 alpha (HIF-1α) can directly regulate the expression of SDF-1α (Ceradini et al., 2004; Du et al., 2012). Tan et al. reported that HDL induced the HIF-1α expression through PI3K/Akt signaling pathways, thereby promoting ischemia-induced angiogenesis (Tan et al., 2014). It has been shown that the expression of HIF-1α can be induced by activating the ERK pathway in ECs (Guo et al., 2019). As the major structural protein of HDL, apoA-I plays a vital role in counteracting the development of AS. Therefore, D-4F or L-4F, an apoA-I mimic peptide (Li Volti et al., 2017; Guo et al., 2020; Peng et al., 2020), may exert similar effects to those of apoA-I. L-4F also prevents myocardial and coronary functional abnormalities in db/db mice through a mechanism involving upregulated adiponectin, pAMPK, and peNOS (Vecoli et al., 2011). Based on the aforementioned, the present study aims to investigate whether D-4F or L-4F promotes the expression of SDF-1α *in vivo* or *in vitro* through the PI3K/Akt/HIF-1α or ERK/HIF-1α signaling pathways.

## Materials and Methods

### Synthesis of the Mimetic Peptide of L-4F

The amino acid sequences of the mimetic peptide of L-4F and scrambled L-4F (sL-4F) (purity as 98%) are as follows: (Ac-DWFKAFYDKVAEKFKEAF-NH2) and (Ac-DWFAKDYFKKAFVEEFAK-NH2), respectively. The amino acid sequences of D-4F and scrambled D-4F (sD-4F) are (Ac-DWFKAFYDKVAEKFKEAF-NH2) and (Ac-DWFAKDYFKKAFVEEFAK-NH2), respectively. They were synthesized by Scilight-Peptide Inc. (Beijing, China), with their structure and purity validated by gas chromatography/ mass spectrometry (GC/MS) method.

### Animals

All mice and experimental procedures were approved by the Animal Experimental Ethics Committee of Weifang Medical University (approval code: 2020SDL142) and performed in accordance with the guidelines for the Care and Use of Laboratory Animals from NIH. Male C57 mice (6 weeks old) were obtained from the Pengyue Company (Shandong, China) and randomly divided into three groups (*n* = 6 for each group): D-4F, sD-4F, and control group, respectively. Mice from D-4F and sD-4F groups were intraperitoneally injected with D-4F and sD-4F [1 mg/kg/day, dissolved in stroke-physiological saline solution (SPSS)] for 9 days, respectively. The time points for peripheral blood collection and dosage of D-4F at 1 mg/kg/day are based on our preliminary work (see Supplementary Figure S1). The control group was treated with the equivalent volume of SPSS.

### Blood, Cells, and Tissue Sampling

Mice were anesthetized by isoflurane inhalation. Peripheral blood was collected from the eye socket vein after overnight fasting at the beginning of the study. Nine days after the injection of D4F (1 mg/kg/day) or sD-4F (1 mg/kg/day) or SPSS, peripheral blood was collected again. Then, the mice were killed with cervical dislocation, and the abdominal aorta and inferior vena cava were separated under a stereomicroscope and stored at −80°C prior to use.

Human umbilical vein endothelial cells (HUVECs) were purchased from the Type Culture Collection of the Chinese Academy of Sciences (Shanghai, China) and cultured in M199 medium [free of fetal bovine serum (FBS), Hyclone, Thermo Fisher Scientific, Waltham, MA, United States] supplemented with 100 U/m penicillin and 100 μg/ml streptomycin under 5% CO_2_ at 37°C.

### Cell Viability

The effects of L-4F on the cell viability of HUVECs were evaluated by 3-(4,5-dimethylthiazol-2-yl)-2,5-diphenyltetrazolium bromide (MTT) method. HUVECs were seeded in a 96-well plate at a density of 1 × 10^4^ cells per well and treated for 24 h with L-4F at various concentrations (final concentrations at 1, 10, 30, 50, and 100 μg/ml, respectively) or sL-4F at 50 μg/ml. Then, 20 μl MTT of 5 mg/ml was employed to each well, and the incubation under the same culture condition lasted for another 4 h before the medium was discarded. Subsequently, each well was added with 150 μl dimethylsulfoxide (DMSO), followed by the determination of absorbance at 450 nm by a microplate spectrophotometer (Multiskan GO, Thermo, United States).

### ELISA Assay

The levels of SDF-1α in mice plasma and the cell culture medium were measured by a commercial ELISA kit (Mlbio, Shanghai, China) according to the producer’s instructions. In brief, L-4F was added to the HUVECs cultured in 24-well plate 2 h after the addition of relevant inhibitors, and the incubation under the same condition was performed for another 24 h. Then, the concentration of SDF-1α was detected by ELISA, and total proteins extracted from HUVECs were quantified by a BCA kit (Solarbio, Beijing, China). The concentration of SDF-1α in the medium was normalized by the total proteins of the HUVECs extract. The levels of SDF-1α were expressed relative to that of control, which was set as 100%.

### Western Blotting Analyses

Proteins levels of HIF-1α, Akt, and ERK were evaluated by Western blotting analyses. Briefly, the abdominal aorta and inferior vena cava were ground by an automatic fast sample grinding instrument at 4°C. On the other hand, L-4F was added to HUVECs cultured in 24-well plate 2 h after the addition of LY294002 at 30 μM ( Sigma, St. Louis, MO, United States) and PD98059 at 20 μM (Sigma, St. Louis, MO, United States), respectively. LY294002 and PD98059 were first dissolved in DMSO to 10 mM and then diluted to the corresponding final working concentration by M199 medium. The incubation under the same condition was performed for different time durations of up to 60 min. Then, total proteins were extracted using cold radio immunoprecipitation assay (RIPA) lysis buffer supplemented with protease and phosphatase inhibitors and quantified using the BCA method. Protein samples of 30 μg were loaded into each well and separated on 8% sodium dodecyl sulfate–polyacrylamide gel electrophoresis (SDS-PAGE), followed by being transferred onto a polyvinylidene fluoride (PVDF) membrane. Under constant shaking, the membrane was blocked using 5% FBS for 2 h at room temperature, incubated with primary monoclonal antibodies of HIF-1α (1: 1,000, Bioss, Beijing, China, RRID: AB_10857933), p-Akt (1: 500, Sigma, St. Louis, MO, United States, RRID:AB_2893426), Akt (1: 500, ImmunoWay, Plano, TX, United States, AB_2893427), p-ERK (1: 100, Santa Cruz, Dallas, TX, United States, RRID: AB_2139990), ERK (1: 100, Santa Cruz, Dallas, TX, United States, RRID: AB_2650548), β-actin (1: 3,000, ImmunoWay, Plano, TX, United States, RRID: AB_2629465), and α-tubulin (1: 3,000, ImmunoWay, Plano, TX, United States, RRID:AB_2893428) at 4°C overnight, rinsed with Tris-buffered saline with Tween 20 (TBST) three times, incubated with horseradish peroxidase (HRP)-conjugated secondary antibodies at room temperature for 2 h, successively. After being washed with phosphate-buffered saline (PBS) three times, the protein bands on the PVDF membrane were visualized using an ECL kit on a chemiluminescence gel imaging system (FluorChem Q, ProteinSimple, CA, United States). β-Actin or α-tubulin was used as an internal reference gene for normalization.

### Statistical Analyses

All data were presented as means ± standard deviation (SD). The data were analyzed using SPSS software (version 18.0, SPSS Inc., Chicago, IL, United States). Differences between two groups were analyzed by unpaired *t*-test after the demonstration of homogeneity of variance with an F-test. Differences between three groups or more were analyzed by one-way ANOVA. Dunnett’s test or Student–Newman–Keuls (SNK) test was used in the *post-hoc* analysis. It was considered statistically significantly different when *p*-value was smaller than 0.05.

## Results

### D-4F Upregulates Protein Levels of SDF-1α in Mice Plasma

**FIGURE 1:**
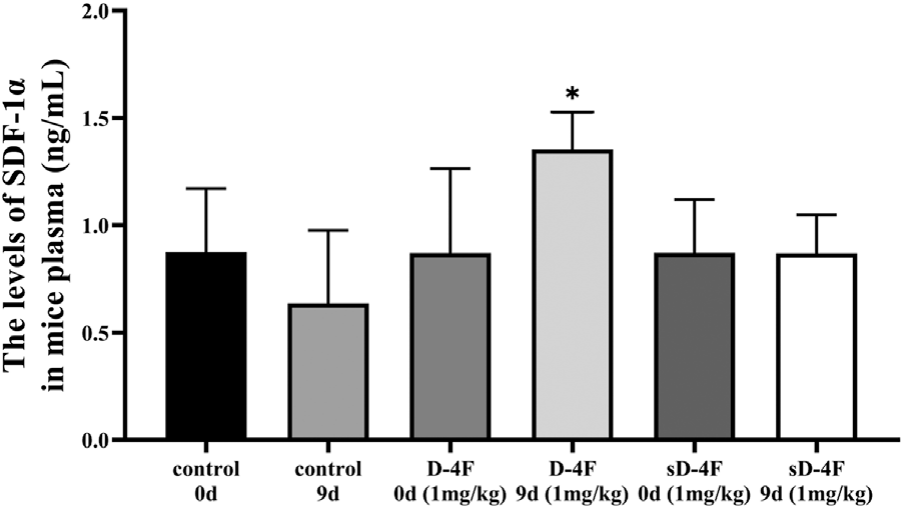
D-4F upregulates the protein levels of SDF-1α in mice plasma. Peripheral blood was collected before and after intraperitoneal injection of D-4F (1 mg/kg/day) or sD-4F (1 mg/kg/day) or equal volume of SPSS for 9 days, *n* = 6, one-way ANOVA, SNK test, *p < 0.05, compared with other groups.

Since D-4F and L-4F demonstrate comparable action and L-4F must be delivered parenterally (Rosenbaum et al., 2015), D-4F and L-4F were therefore employed for *in vivo* and *in vitro* investigations in the present work, respectively. The peripheral blood of mice was collected from day 0 to 11 after D-4F injection. The effects of D-4F on the levels of SDF-1α in mice plasma were evaluated by ELISA, and the results are shown in Figure 1. It can be learned that D-4F at a concentration of 1 mg/kg/day for 9 days significantly upregulated the levels of SDF-1α in mice plasma compared with control group or the D-4F group before injection.

### D-4F Upregulates Protein Levels of HIF-1α, Phosphorylated Akt, and Phosphorylated ERK in the Abdominal Aorta and Inferior Vena Cava of Mice

**FIGURE 2:**
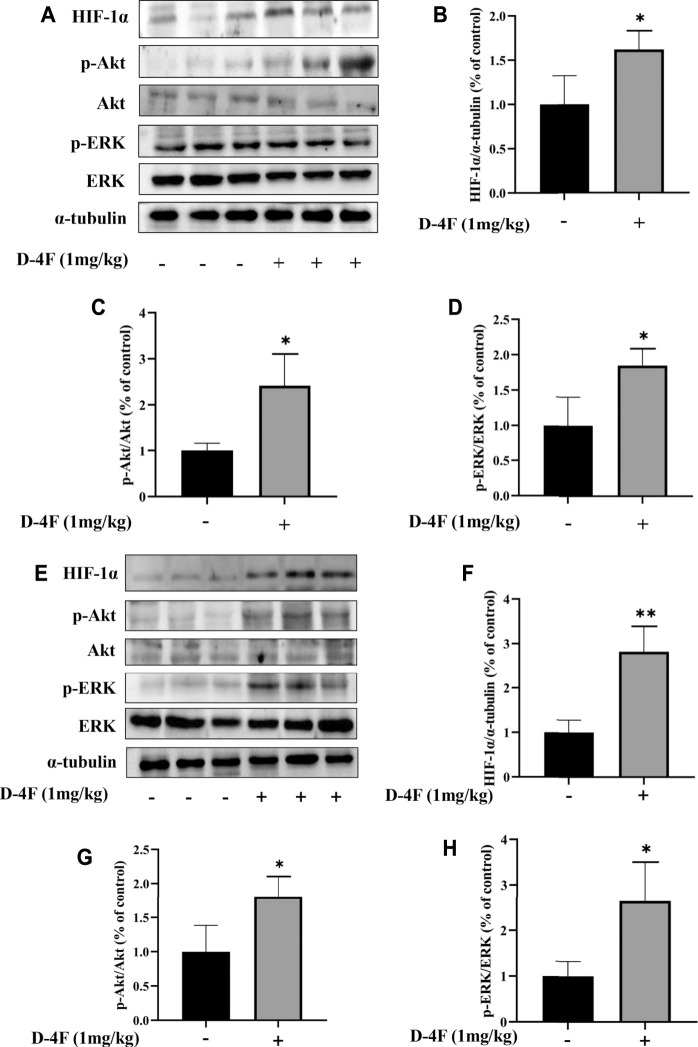
D-4F upregulates protein levels of HIF-1α, p-Akt, and p-ERK in mice. **(A–D)** Abdominal aorta. **(E–H)** Inferior vena cava. Mice were treated with or without D-4F at 1 mg/kg/day for 9 days, *n* = 6, unpaired *t*-test, *p < 0.05, **p < 0.01, compared with the group without the addition of D-4F.

**FIGURE 3:**
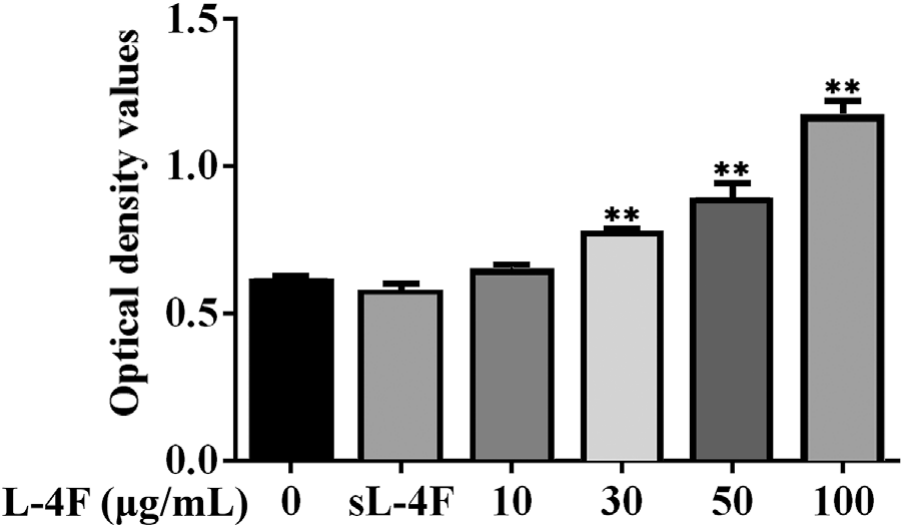
L-4F improves cell viability of HUVECs. Cells were treated for 24 h with L-4F at various concentrations (final concentrations at 0, 10, 30, 50, and 100 μg/ml, respectively) or sL-4F at 50 μg/ml, *n* = 6, one-way ANOVA, SNK test, **p < 0.01, compared with the group at 0 μg/ml.

**FIGURE 4:**
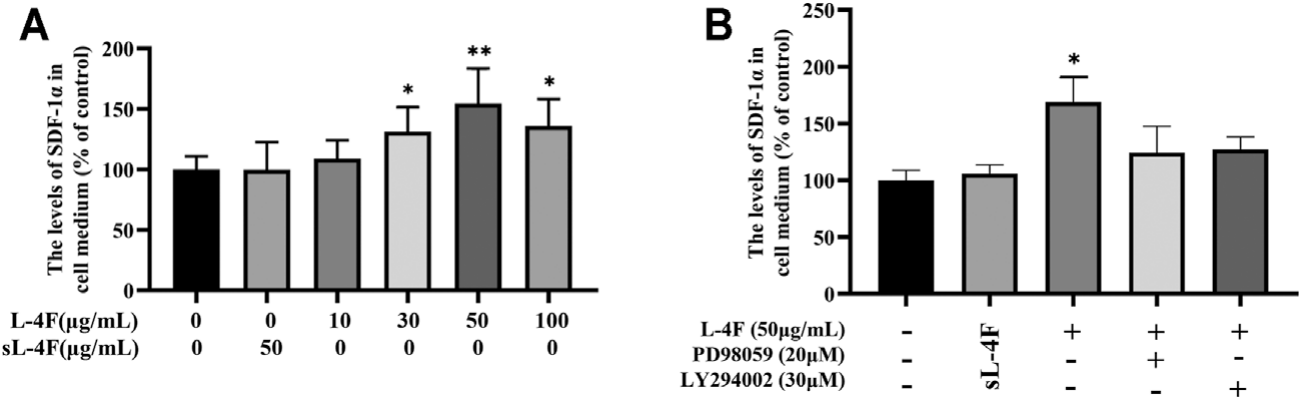
L-4F upregulates the protein levels of SDF-1α in HUVECs. **(A)** Cells were treated for 24 h with L-4F at various concentrations (final concentration at 0, 10, 30, 50, and 100 μg/ml, respectively) or sL-4F at 50 μg/ml (*n* = 6, one-way ANOVA, Dunnett’s test). **(B)** Cells were pretreated with and without relevant specific inhibitors for 2 h prior to the treatment with L-4F or sL-4F at 50 μg/ml for 24 h (*n* = 6, SNK test). *p < 0.05, **p < 0.01, compared with control.

The effects of D-4F on protein levels of HIF-1α, p-Akt, and p-ERK in the abdominal aorta and inferior vena cava were investigated by Western blotting analyses. As shown in Figure 2, D-4F significantly upregulates the levels of HIF-1α, p-Akt, and p-ERK in abdominal aorta and inferior vena cava.

### L-4F Improves the Cell Viability of HUVECs

Next, we employed HUVECs as an *in vitro* cellular model to investigate the mechanism underlying the promotive effects of 4F on the levels of SDF-1α. To determine an appropriate working concentration of L-4F, its effects on the cell viability of HUVECs were evaluated by MTT method. As shown in Figure 3, L-4F at a concentration not higher than 100 μg/ml exerts no significant cytotoxic effects on HUVECs, nor does the sL-4F at 50 μg/ml. L-4F improves the cell viability of HUVECs in a concentration-dependent manner.

### L-4F Upregulates Protein Levels of SDF-1α in Cell Medium

The effects of L-4F on the levels of SDF-1α in cell medium were determined by ELISA, and the results are shown in Figure 4. It could be learned that L-4F at a concentration of ≤50 μg/ml upregulates the levels of SDF-1α in a concentration-dependent manner when compared with the control group. Therefore, L-4F at 50 μg/ml was employed in the subsequent experiments. The specific inhibitors of LY294002 and PD98059 pretreatment significantly suppress the levels of SDF-1α promoted by L-4F. There is no significant difference between the 50 μg/ml sL-4F group and the control.

### L-4F Upregulates the Protein Levels of HIF-1α, p-Akt, and p-ERK in HUVECs

**FIGURE 5:**
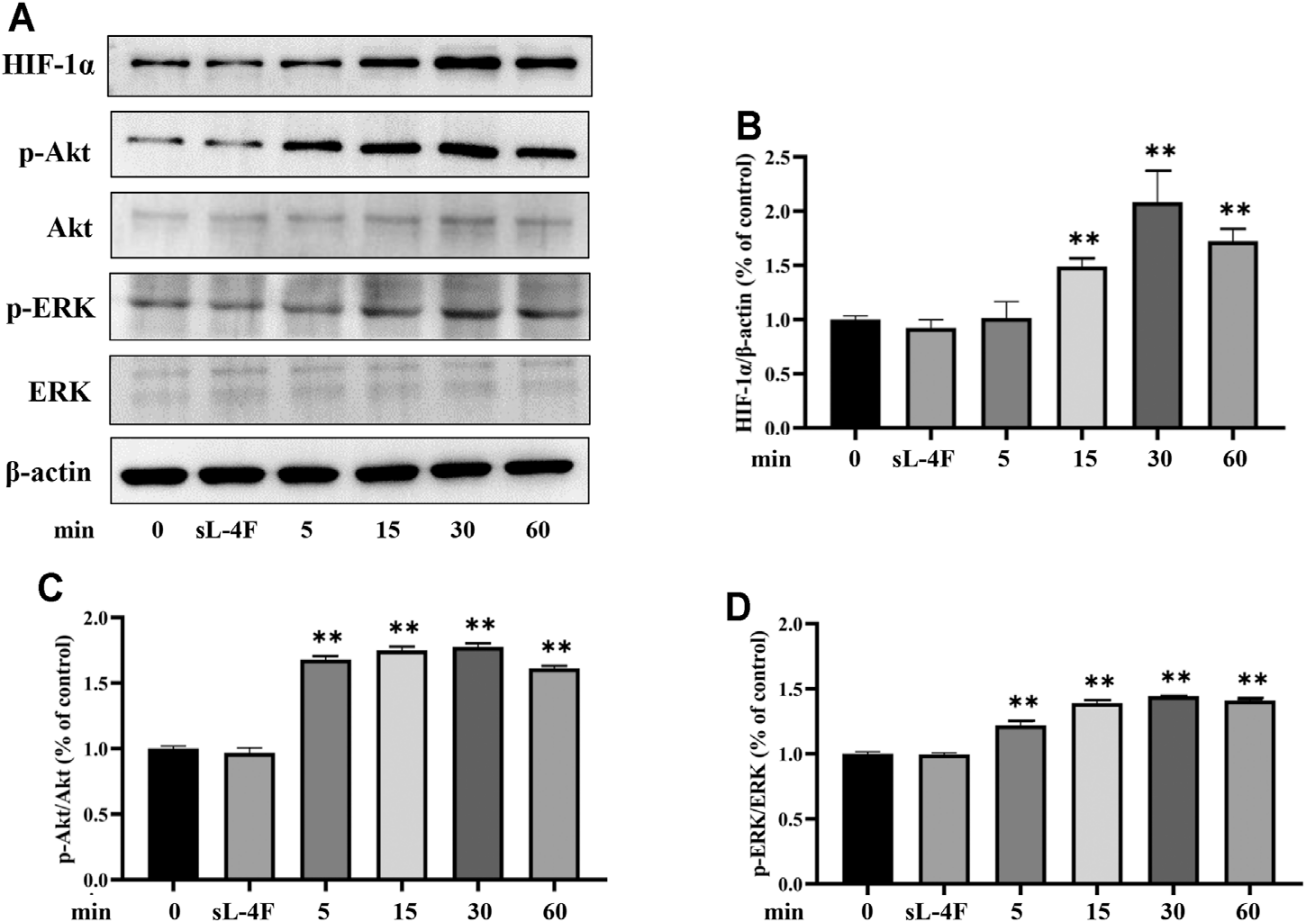
**(A)** Representative Western blot images. L-4F upregulates protein levels of HIF-1α **(B)**, p-Akt **(C)**, and p-ERK **(D)** in HUVECs at different time points. Cells were treated with L-4F (50 μg/ml) for 0, 5, 15, 30, and 60 min, respectively, or sL-4F at 50 μg/ml for 30 min, *n* = 3, one-way ANOVA, Dunnett’s test, **p < 0.01, compared with control.

**FIGURE 6:**
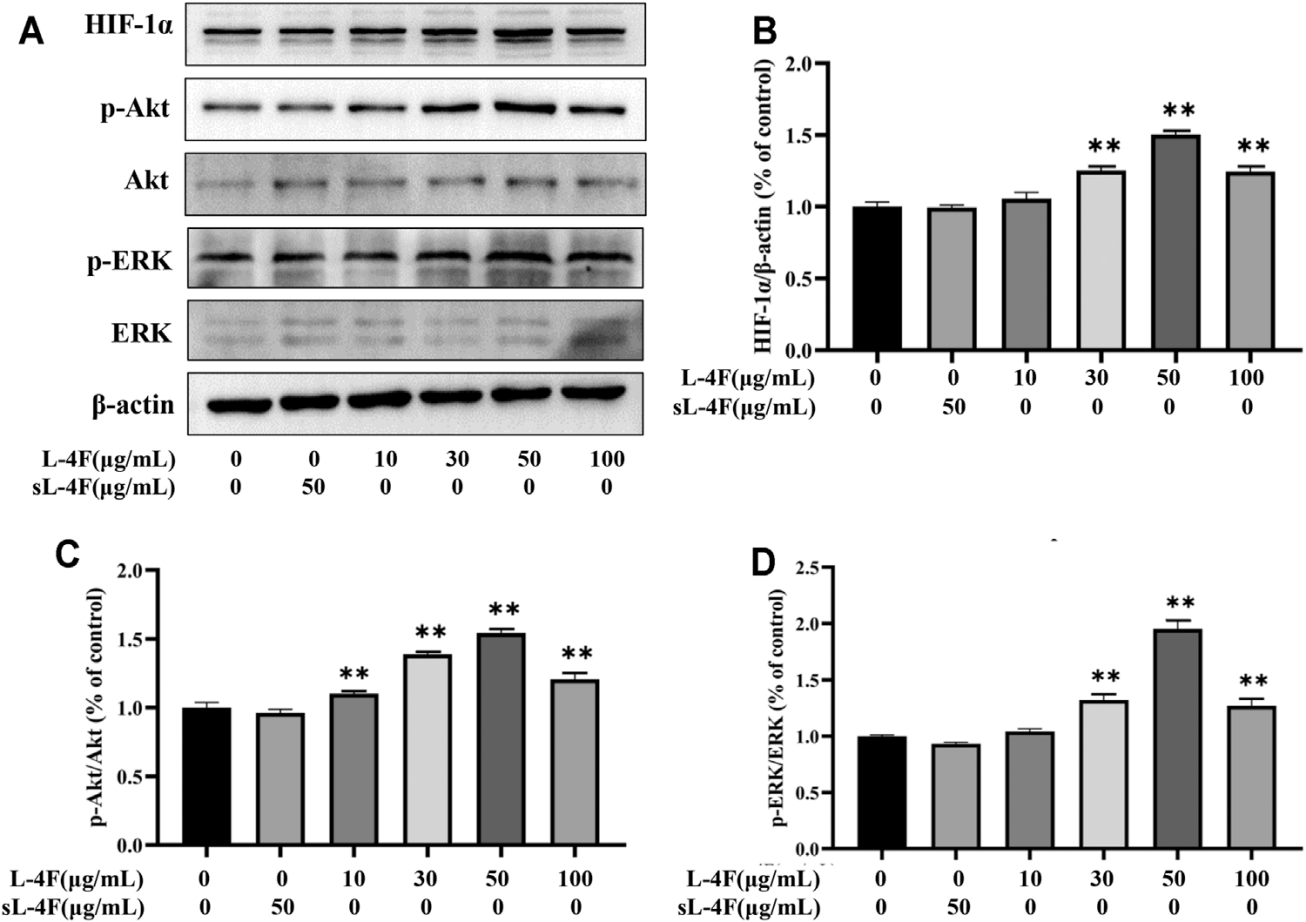
**(A)** Representative Western blot images. L-4F at different concentrations upregulates protein levels of HIF-1α **(B)**, p-Akt **(C)**, and p-ERK **(D)** in HUVECs. Cells were treated with L-4F at 0, 10, 30, 50, and 100 μg/ml, respectively, or sL-4F at 50 μg/ml for 30 min, *n* = 3, one-way ANOVA, Dunnett’s test, *p < 0.05, **p < 0.01, compared with control.

The effects of L-4F at different time points and concentrations on protein levels of HIF-1α, p-Akt, and p-ERK in HUVECs were investigated by Western blotting analyses. The results are presented in Figures 5 and 6, respectively. Compared with control, L-4F treatment for 30 min exerts the most significant effects on protein levels of HIF-1α, p-Akt, and p-ERK (Figures 5A–D). L-4F upregulates the protein levels of HIF-1α, p-Akt, and p-ERK in a concentration-dependent manner at a concentration of ≤50 μg/ml in comparison with control (Figures 6A–D).

### Pretreatment of LY294002 and PD98059 Suppresses Protein Levels of HIF-1α, p-Akt, and p-ERK Induced by L-4F in HUVECs

**FIGURE 7:**
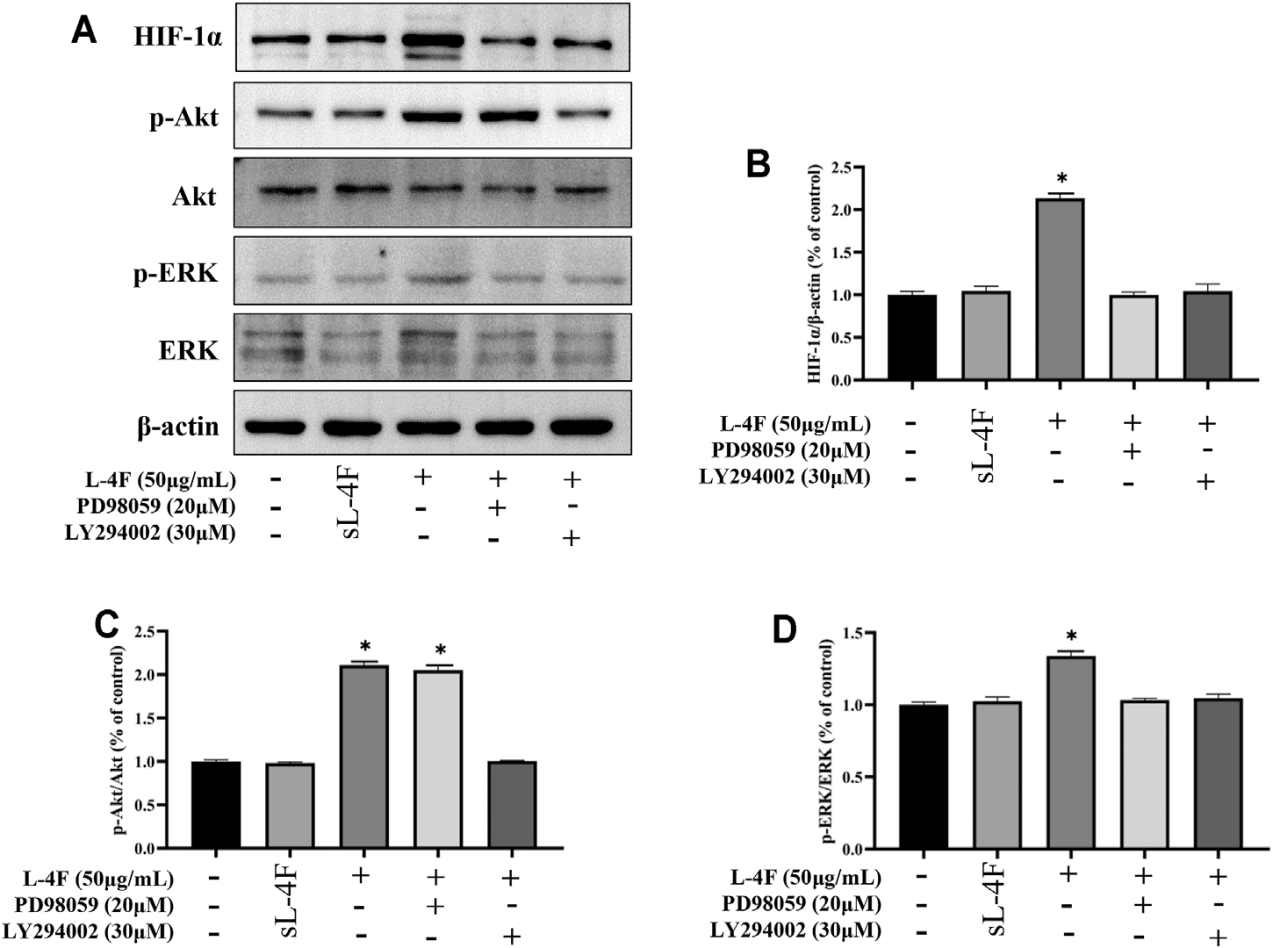
**(A)** Representative Western blot images. Pretreatment of LY294002 and PD98059 suppresses protein levels of HIF-1α **(B)**, p-Akt **(C)**, and p-ERK **(D)** induced by L-4F in HUVECs. Cells were pretreated with and without relevant specific inhibitors for 2 h prior to the treatment with L-4F or sL-4F at 50 μg/ml for 30 min, *n* = 3, one-way ANOVA, SNK test, **p < 0.01, compared with other groups.

The effects of pretreatment of specific inhibitors of LY294002 and PD98059 on protein levels of HIF-1α, p-Akt, and p-ERK induced by L-4F in HUVECs were studied by Western blotting analysis (Figures 7A–D). It can be found that the upregulated protein levels of HIF-1α and p-ERK caused by L-4F can be significantly suppressed by LY294002 and PD98059 (Figures 7B and D). However, the elevated levels of p-Akt by L-4F could not be significantly inhibited by PD98059, but LY294002 (Figure 7C), also suggesting ERK as a downstream molecule of PI3K signaling pathway in the present work.

## Discussion

In consideration of the critical role of EPCs in neovascularization and resistance to ischemic lesions as aforementioned, it is of great importance to investigate potential means to activate functional EPCs. SDF-1α has been well demonstrated to be involved in the mobilization, trafficking, and homing of EPCs derived from the bone marrow to ischemic lesions through its specific receptor of CXCR4 (Ceradini et al., 2004). Therefore, SDF-1α seems to play a vital protecting role against cardiovascular disease. The present work shows that the apoA-I mimic peptide of 4F can effectively induce the expression of SDF-1α in HUVECs and plasma in mice, which may be through the signaling pathway of PI3K/Akt/ERK/HIF-1α.

Numerous studies have exhibited that the expression of SDF-1α is regulated by the transcription factor of HIF-1α *in vivo* and *in vitro* (such as ECs), which is consistent with the present work (Loh et al., 2009; Zan et al., 2015). Generally, HIF-1α is kept at extremely low levels and can be induced by hypoxia (Huang et al., 1998). Recently, HIF-1α was reported to be induced by many other factors such as insulin and angiotensin II, and the activation of PI3K/Akt and ERK1/2 signaling pathways even under normoxic conditions (Pagé et al., 2002; Hartmann et al., 2008; Tan et al., 2014). The elevated HIF-1α can mediate multiple processes, including angiogenesis, and regulate a lot of target genes such as SDF-1α (Semenza, 2003). As one of the numerous downstream effectors of HIF-1α, SDF-1α can be transcriptionally regulated through binding the hypoxia response elements within its promoter to HIF-1α (Ceradini et al., 2004). It should be noted that HIF-1α improves the EPCs function during the process of angiogenesis by upregulating many factors such as VEGF (Zan et al., 2015).

**FIGURE 8:**
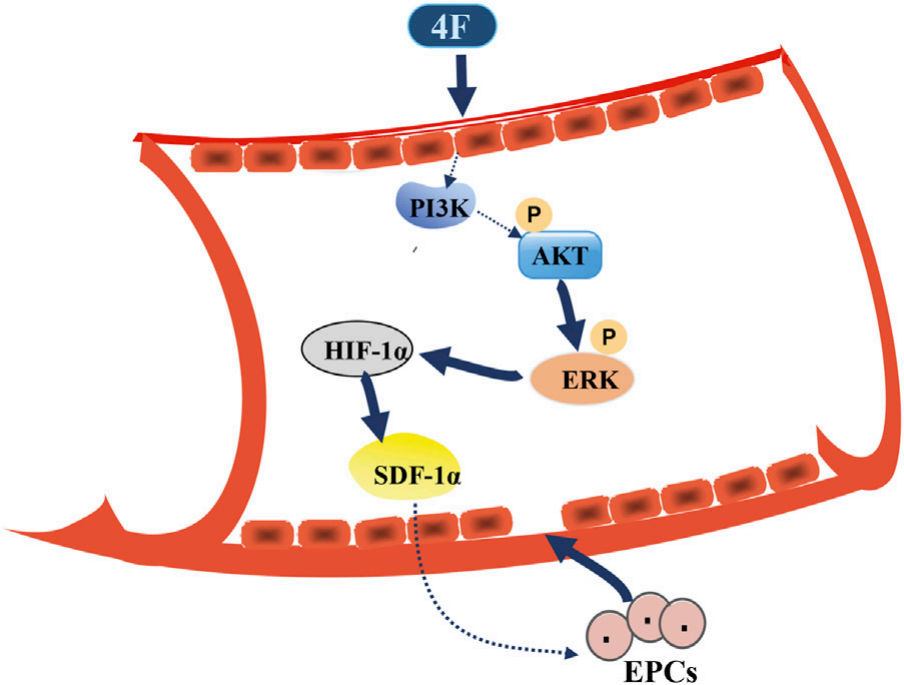
Schematic diagram for 4F promoting SDF-1α expression in vascular endothelial cells. 4F can upregulate the levels of HIF-1α in mice and endothelial cells by activating PI3K/Akt/ERK signaling pathway and thus promoting the level of SDF-1α. Elevated SDF-1α in blood circulation may mobilize EPCs in the bone marrow to migrate to the site of vascular intimal injury where EPCs differentiate into mature ECs and participate in the repair of vascular endothelial injury.

The expression of HIF-1α has been reported to be closely related to the activation of PI3K/Akt signaling pathway, which widely participates in the migration and localization of numerous cells (Yu et al., 2010; Zheng et al., 2007). PI3K can activate its downstream kinases of Akt and mTOR (Fresno Vara et al., 2004). Moreover, the *trans*-activation activity and stability of HIF-1α can also be significantly elevated by the signaling pathway of ERK1/2 MAPK through directly phosphorylating its C-terminal domain (Minet et al., 2000; Sang et al., 2003). These two signaling pathways work mainly dependent on the kinase of p70 S6 to ultimately enhance the translation rate of HIF-1α from mRNA to protein *via* the initiating a cascade of events (Semenza, 2003). In addition to the upregulation of SDF-1α, as evidenced in the present work, these two signaling pathways also involve the regulation of the growth, survival, and migration of ECs in angiogenesis (Gates et al., 2007; Karar and Maity, 2011). Interestingly, it can be learned from Figures 6 and 8 that ERK functions as a downstream molecule of PI3K signaling pathway. MEK⁄ERK has been demonstrated to be regulated by Akt1 *via* the PAK pathway (Lee et al., 2011). The results suggested that 4F induces the expression of SDF-1α in HUVECs and mice through PI3K/Akt/ERK/HIF-1α signaling pathway.

The overexpression of apoA-I has been found to possess the capability to elevate the number of circulating EPCs, which plays a key part in the process of neovascularization induced by ischemia (Feng et al., 2009). These effects rely on the same signaling pathways of PI3K/Akt and mitogen-activated protein kinase (MAPK), which have been shown to be capable of driving the pathway of HIF-1α/VEGF (Miura et al., 2003; Mineo and Shaul, 2007). As a mimic peptide of apoA-I, 4F significantly activates the PI3K/Akt/ERK/HIF-1α signaling pathway in the present study. Therefore, 4F may exert its protective effects against AS and the promotion of neovascularization through a similar mechanism to apoA-I.

Although the activation of HIF-1α acts as an adaptive response to hypoxia for cells, its prolonged activation is deleterious to hypoxic cells, and overexpressed HIF-1α has been reported to be associated with tumor growth, cancer metastasis, and poor prognosis, and tumor chemotherapy resistance (Shoshani et al., 2002; Goda et al., 2003; Wei et al., 2013; Doktorova et al., 2015; Zhang et al., 2015). Moreover, elevated SDF-1α should be evaluated cautiously, since it could promote the aggregation of platelet (Abi-Younes et al., 2000). Therefore, further studies are needed for optimizing the expression of SDF-1α probably through the employment of an inducible vector. In addition, HIF-1α-independent mechanisms underlying the expression of SDF-1α should be further investigated (Lerman et al., 2010).

It has been reported that HIF-1α can mobilize EPCs to the site of vascular intimal injury and promote the repair of vascular endothelial injury through promoting the level of SDF-1α *in vivo* (Ceradini et al., 2004; Hiasa et al., 2004). One of our previous papers also suggests that in C57 mice fed by high-fat diet, reverse-D-4F (demonstrating similar function to 4F) can increase the number of EPCs and SDF-1α levels (Yang et al., 2015). However, there are still some limitations in the present work. First, the biological effects of elevated SDF-1α induced by 4F *in vivo* and the underlying mechanisms have not been deeply studied in the present study. Second, whether 4F mobilizes EPCs to the damaged vascular intima and inhibits the occurrence and development of atherosclerosis *in vivo* is not investigated in the present work. Next, we will employ conditional gene knockout animals, inhibitors, antibody intervention, etc. to investigate the underlying mechanisms aforementioned *in vivo*.

In conclusion, 4F can promote the expression of SDF-1α in ECs and mice through PI3K/Akt/ERK/HIF-1α signaling pathway. The improved levels of SDF-1α in blood may mobilize EPCs from the bone marrow and tissues to the injured intima to restore vascular endothelium.

## Data Availability

The original contributions presented in the study are included in the article/Supplementary Material. Further inquiries can be directed to the corresponding authors.
