# Supplementary material for: An ApoA-I Mimic Peptide of 4F Promotes SDF-1α Expression in Endothelial Cells Through PI3K/Akt/ERK/HIF-1α Signaling Pathway
Source: Front Pharmacol. 2022 Jan 17;12:760908. doi: 10.3389/fphar.2021.760908 (PMC8801807; doi:10.3389/fphar.2021.760908)

Raw data for WB of figure 2 A

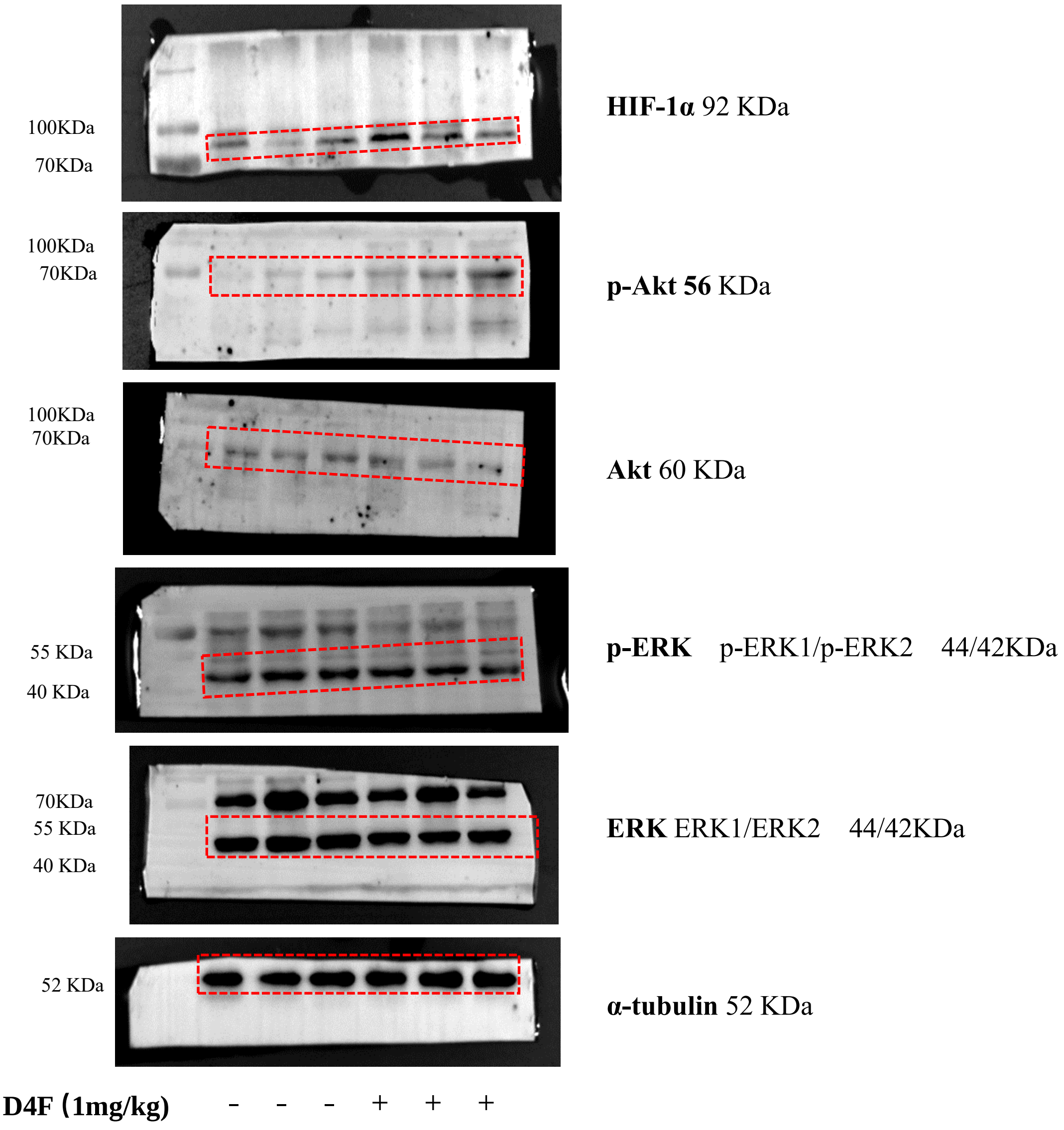

Raw data for WB of figure 2 E

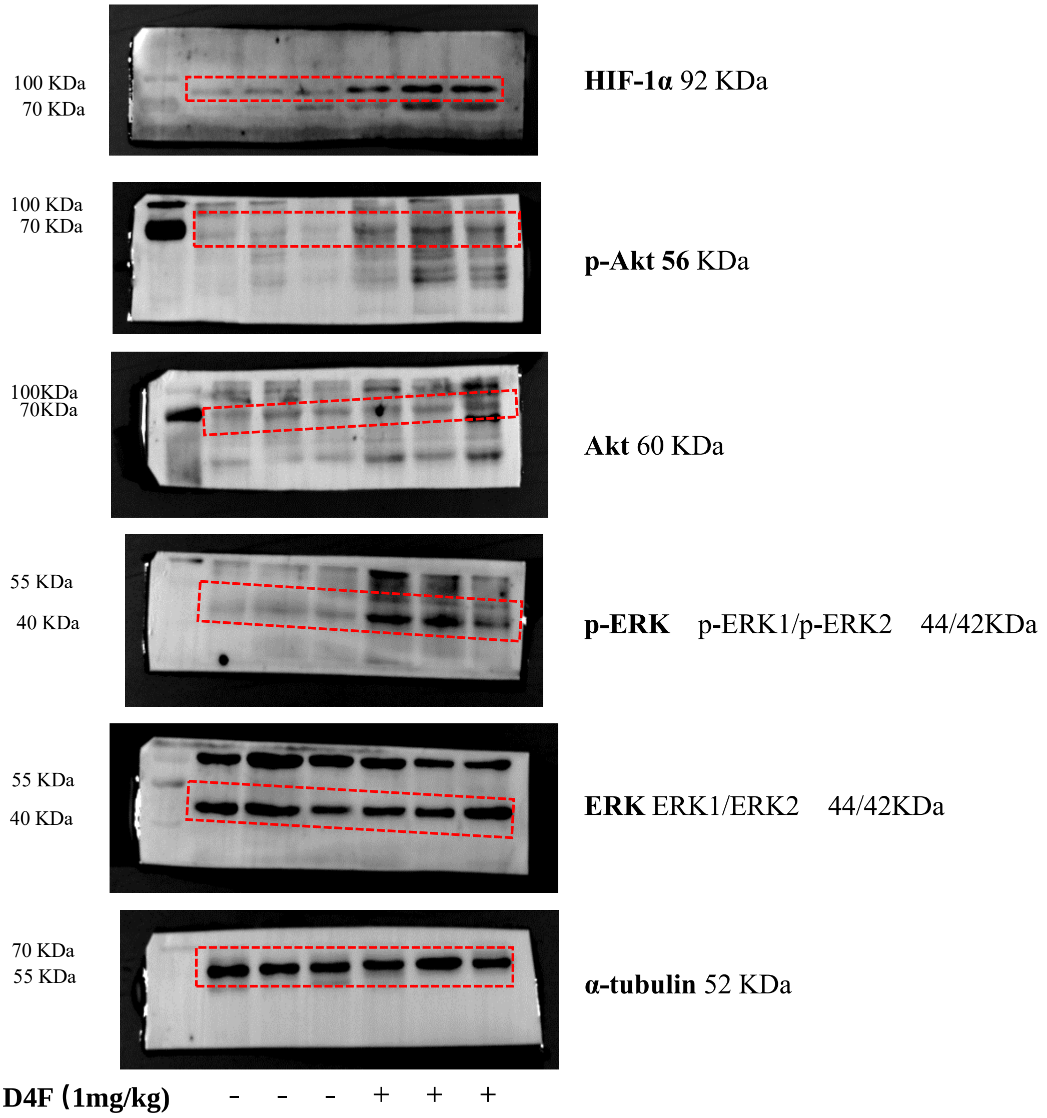

Raw data for WB of figure 5 A

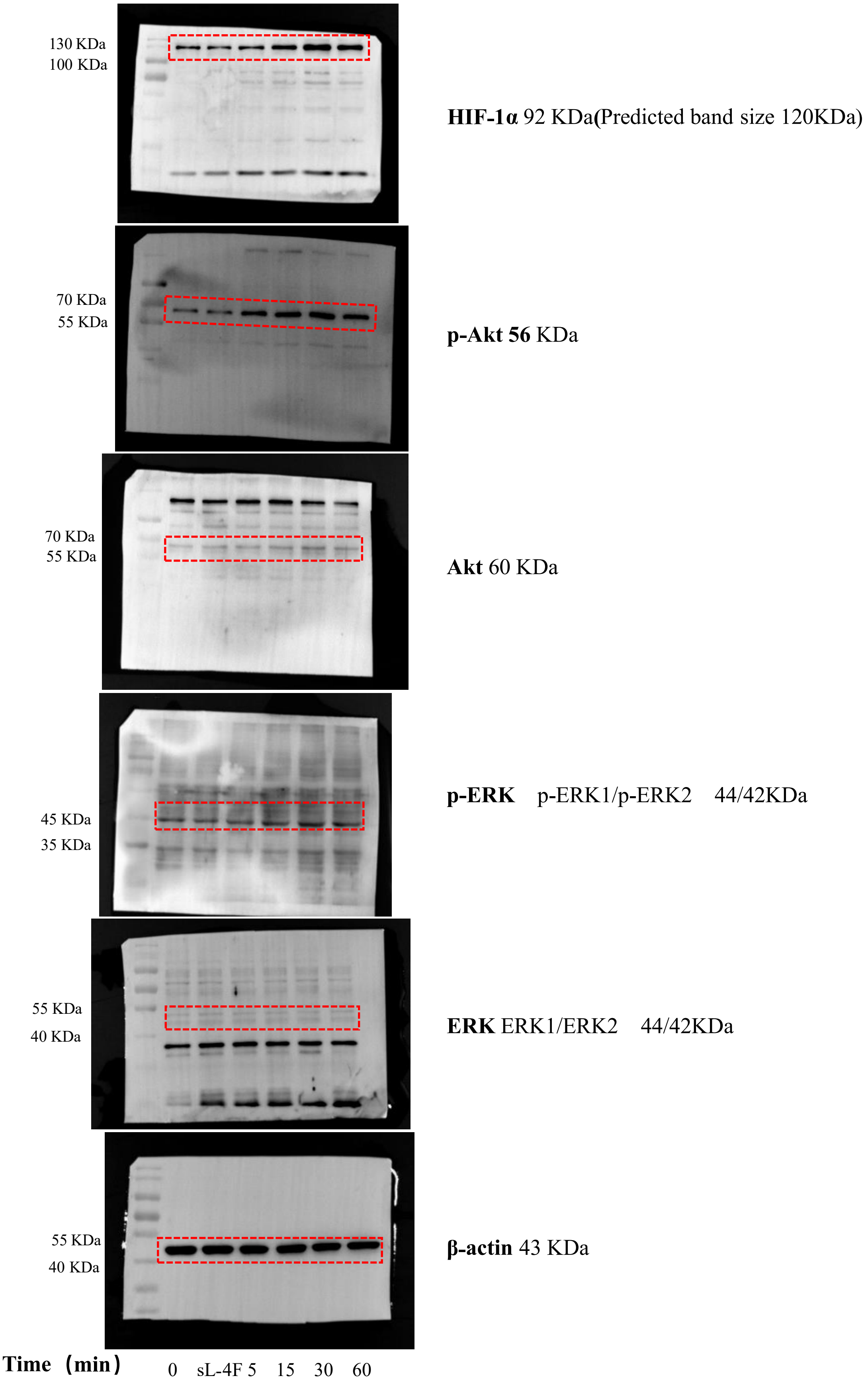

Raw data for WB of figure 6 A

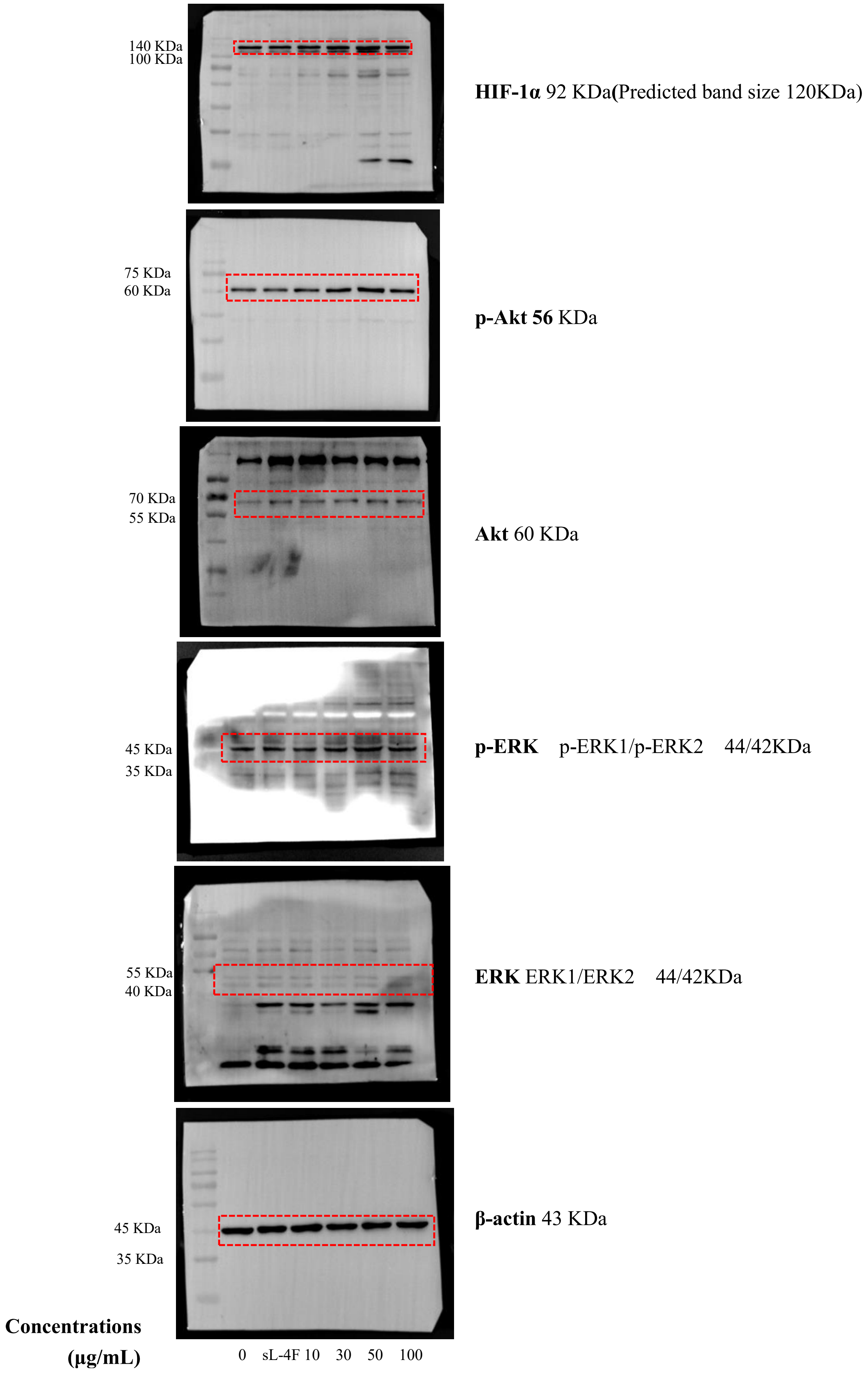

Raw data for WB of figure 7 A

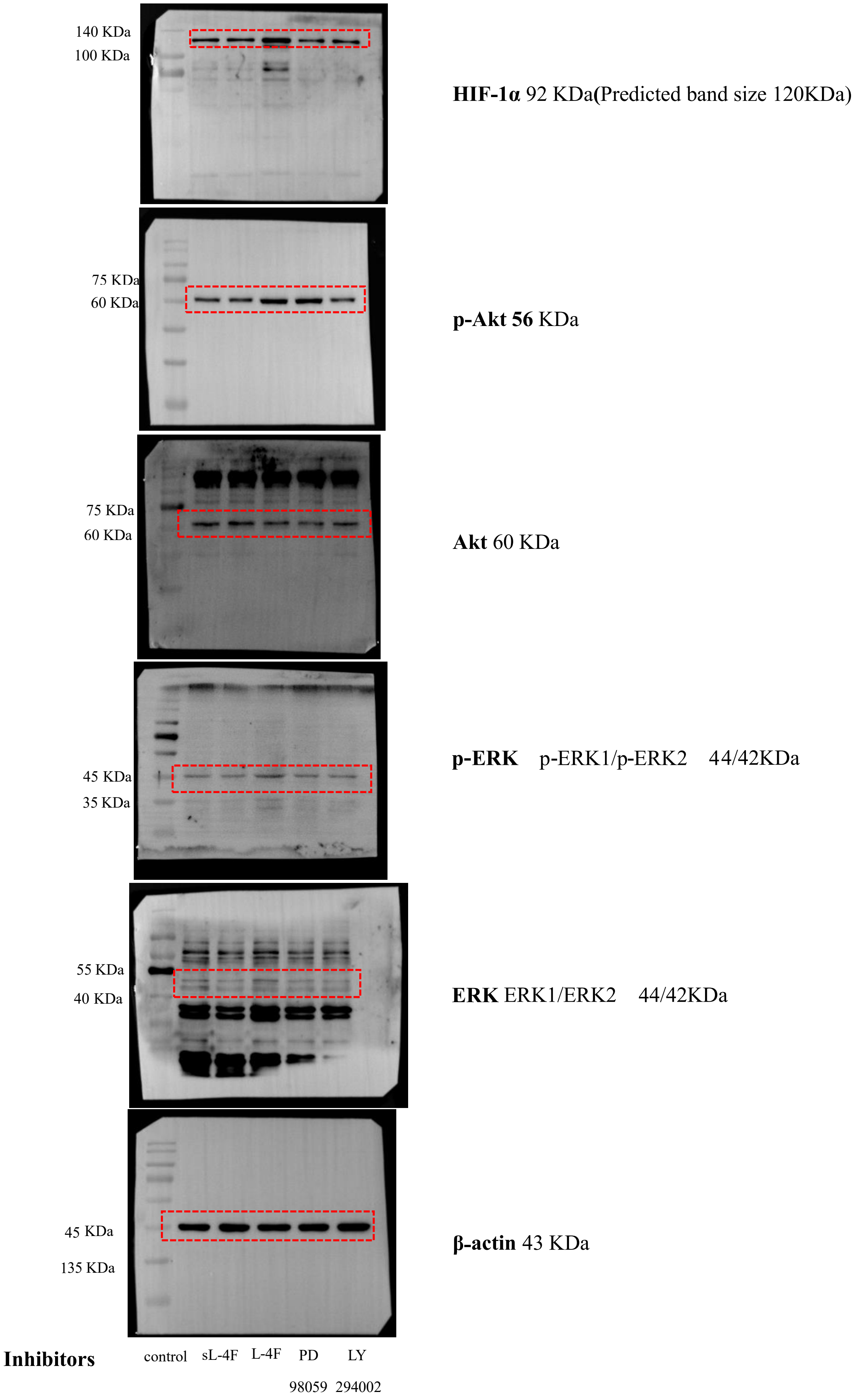

Raw data for WB of Supplementary Figure 2 A

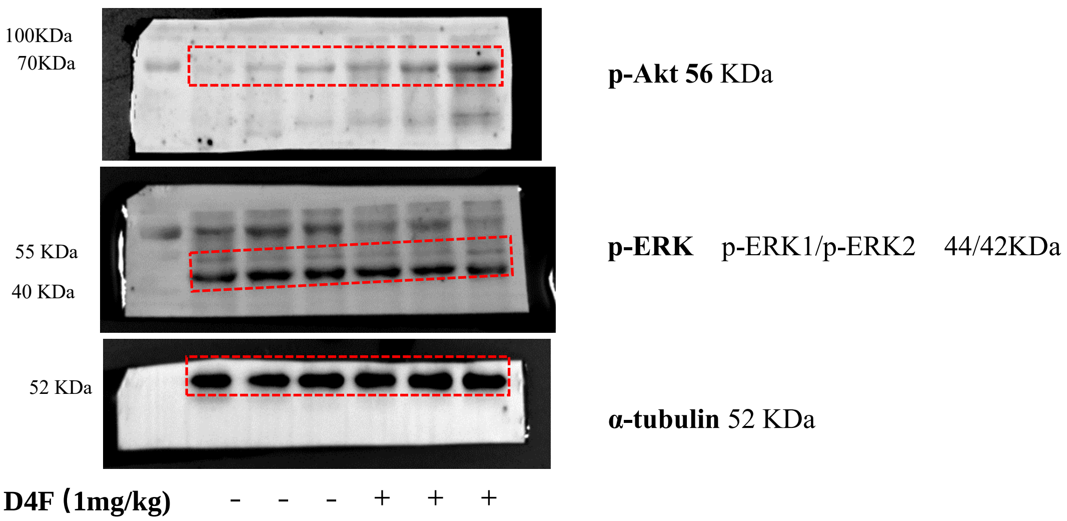

Raw data for WB of Supplementary Figure 2 D

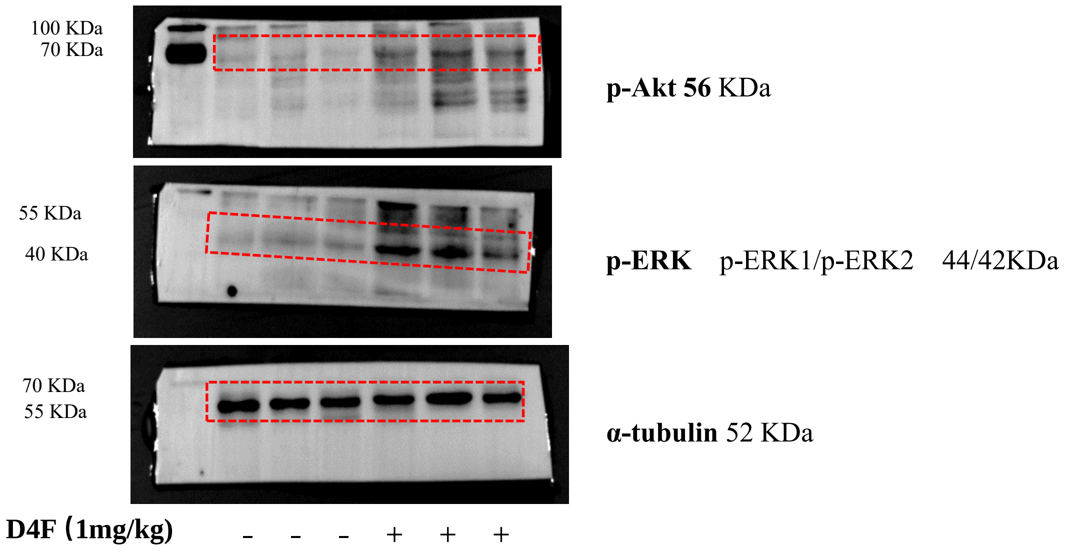

Raw data for WB of Supplementary Figure 3 A

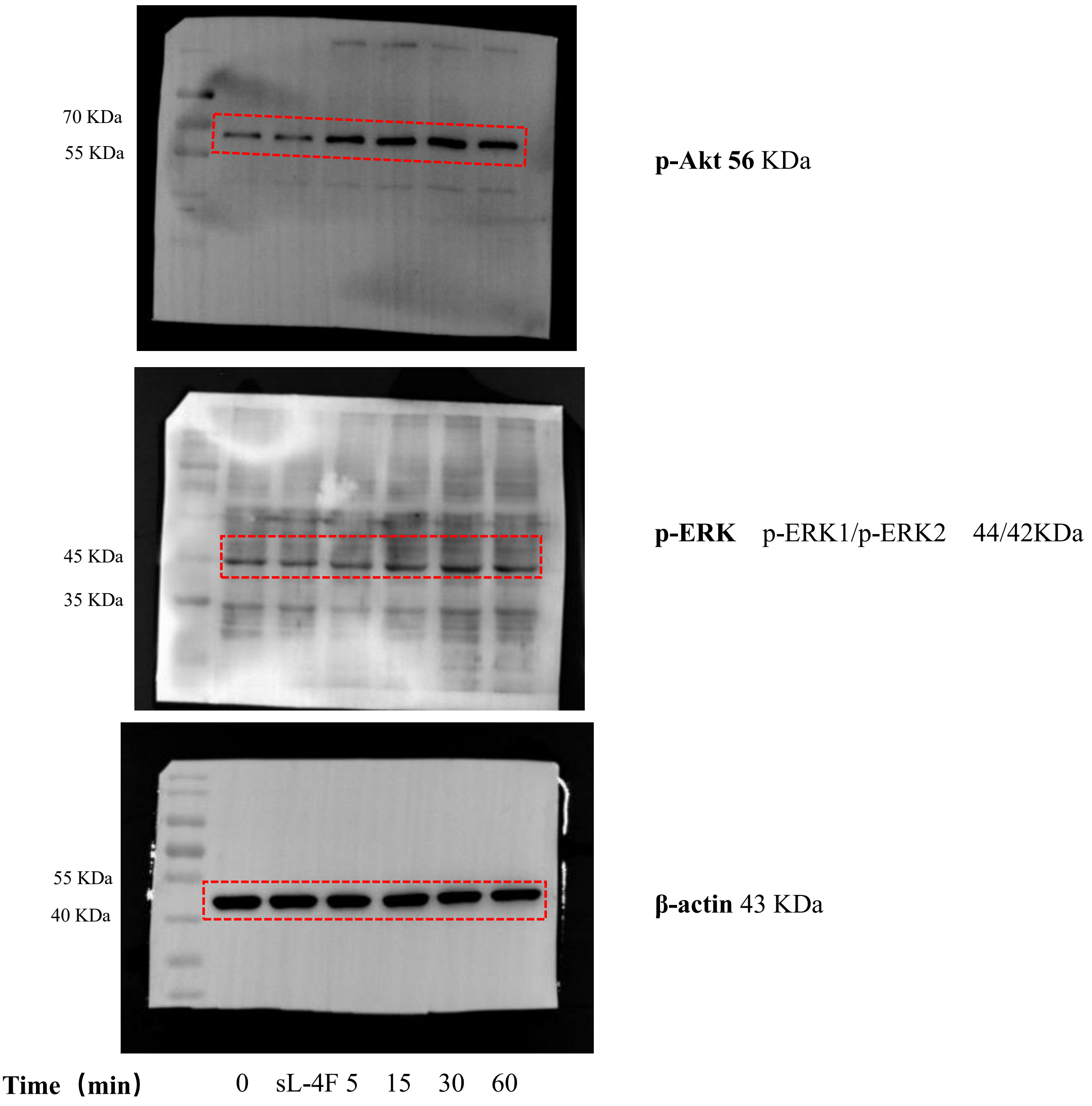

Raw data for WB of Supplementary Figure 4 A

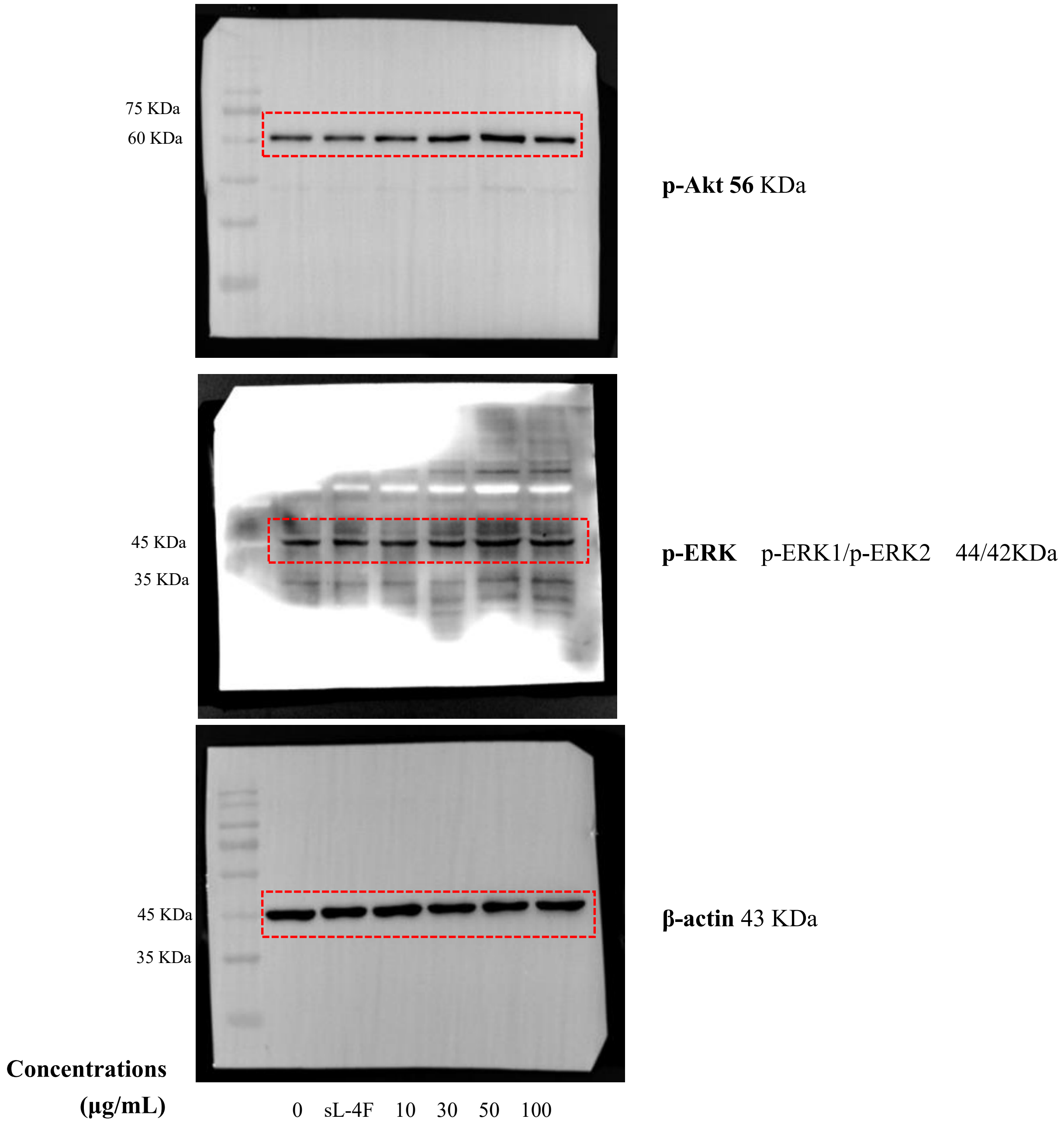

Raw data for WB of Supplementary Figure 5 A

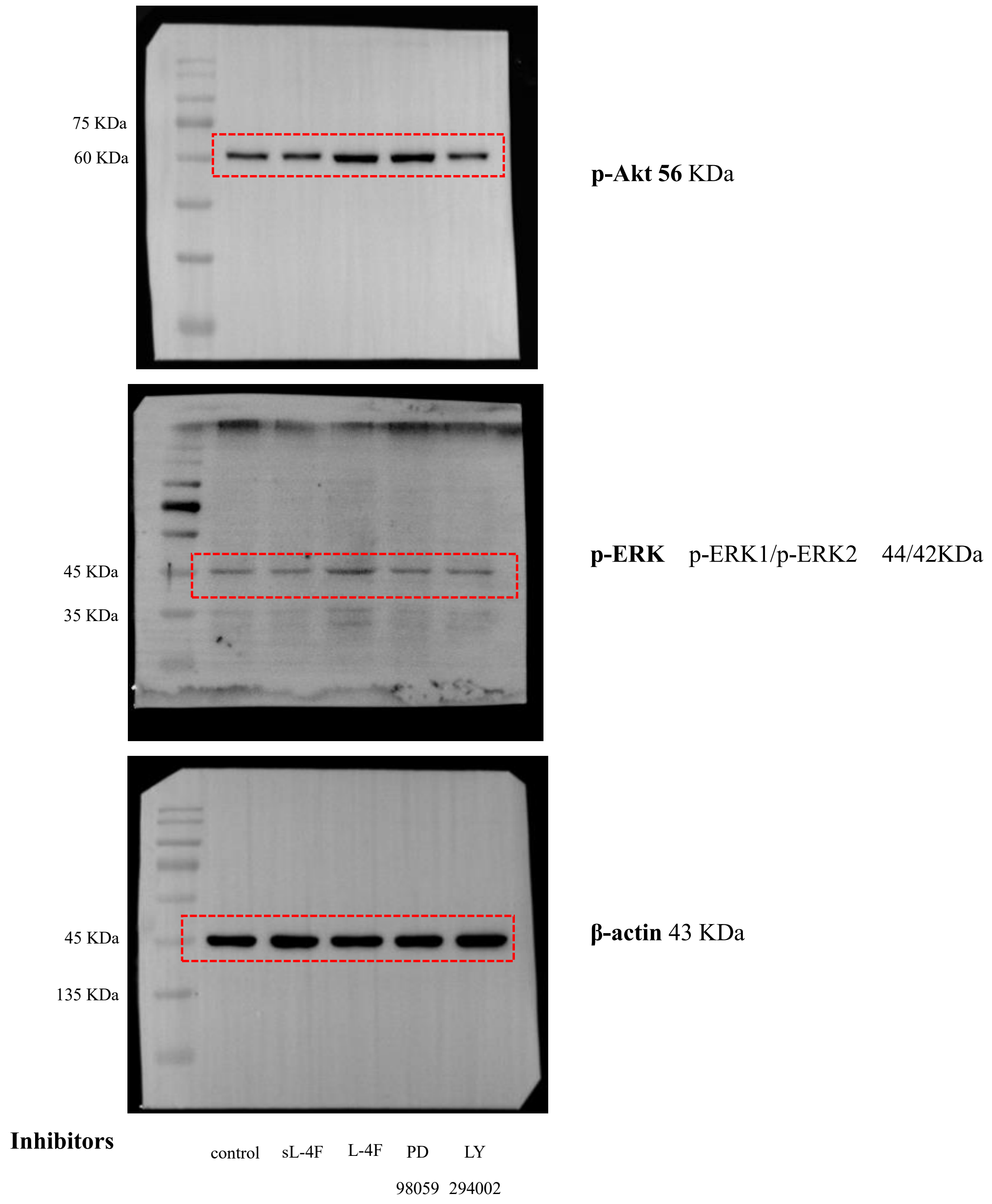

Supplement: Supplementary file 1 [file DataSheet1.pdf]
