# Supplementary material for: An ApoA-I Mimic Peptide of 4F Promotes SDF-1α Expression in Endothelial Cells Through PI3K/Akt/ERK/HIF-1α Signaling Pathway
Source: Front Pharmacol. 2022 Jan 17;12:760908. doi: 10.3389/fphar.2021.760908 (PMC8801807; doi:10.3389/fphar.2021.760908)
Supplement: Supplementary file 2 [file DataSheet2.DOCX]

Supplementary Material

# Supplementary Figures


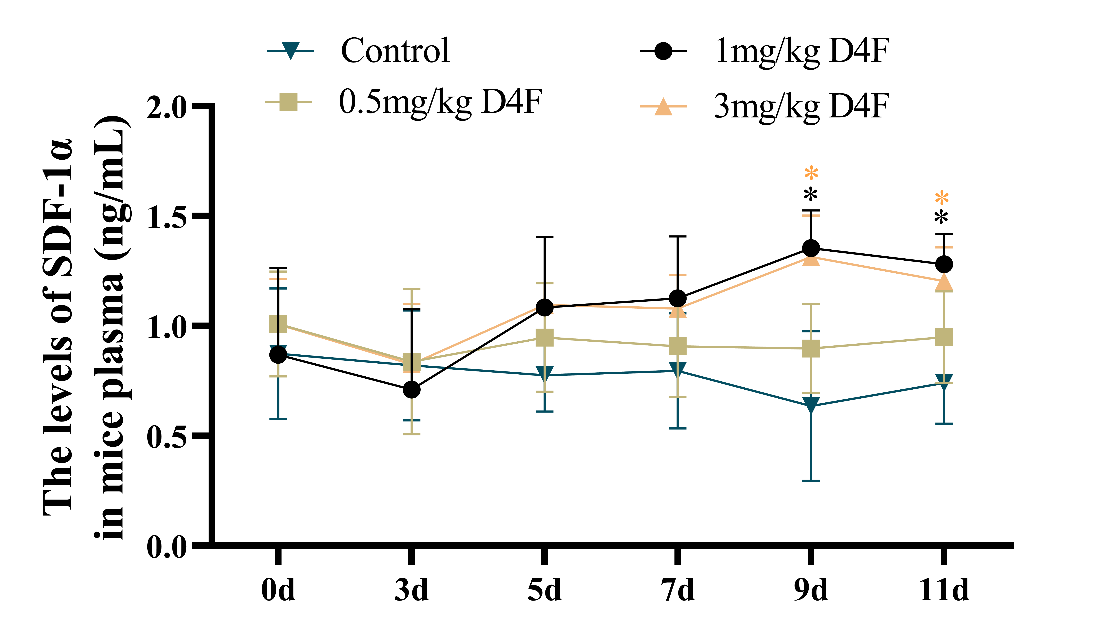


**Supplementary Figure 1.** SDF-1α level significantly increases on the 9th day in mice after D-4F injection at 1mg/kg/day. Peripheral blood was collected at 0d, 3d, 5d, 7d, 9d, and 11d after intraperitoneal injection of D-4F at various concentrations of 0, 0.5, 1, and 3 mg/kg/day for 11 days. n=6, one-way ANOVA, Dunnett's test, *P<0.05, compared with control.

**
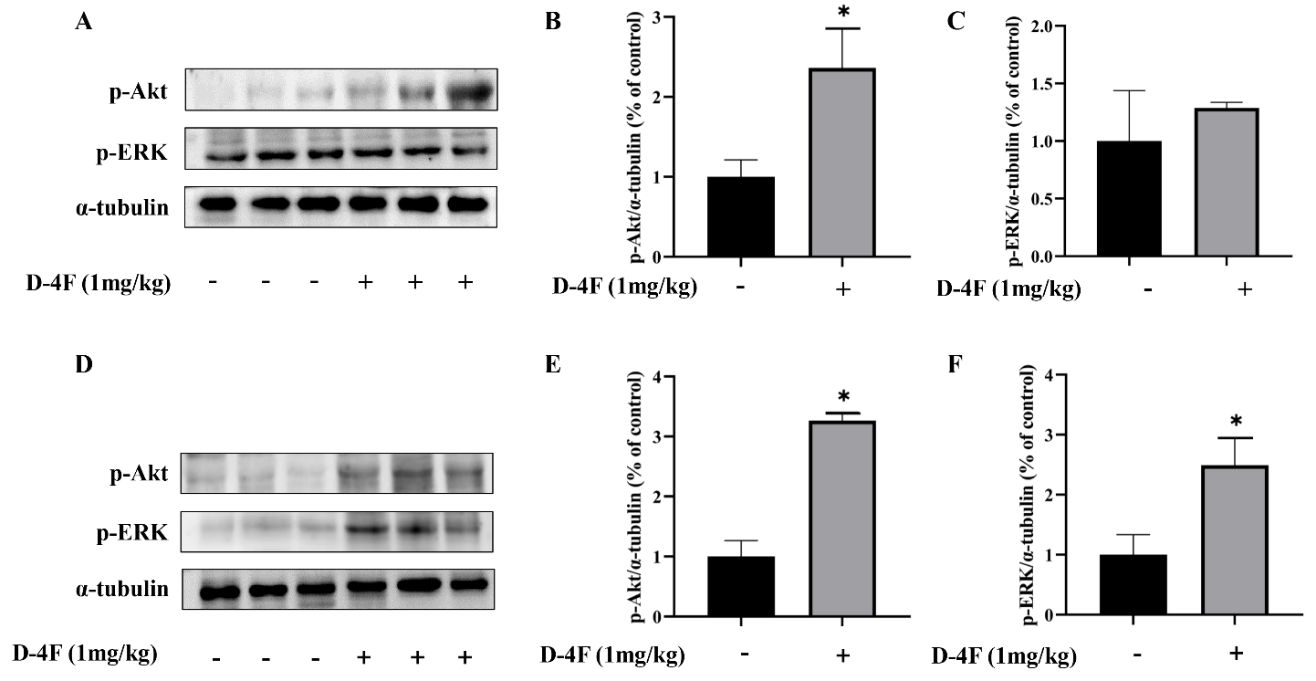
**

**Supplementary Figure 2.** Protein levels of p-Akt and p-ERK in mice abdominal aorta (A-C) and inferior vena cava (D-F). Mice were treated with or without D-4F at 1mg/kg/day for 9 days. n=6, unpaired t test, normalized to α-tubulin, *P<0.05 compared with the group without the addition of D-4F.

**
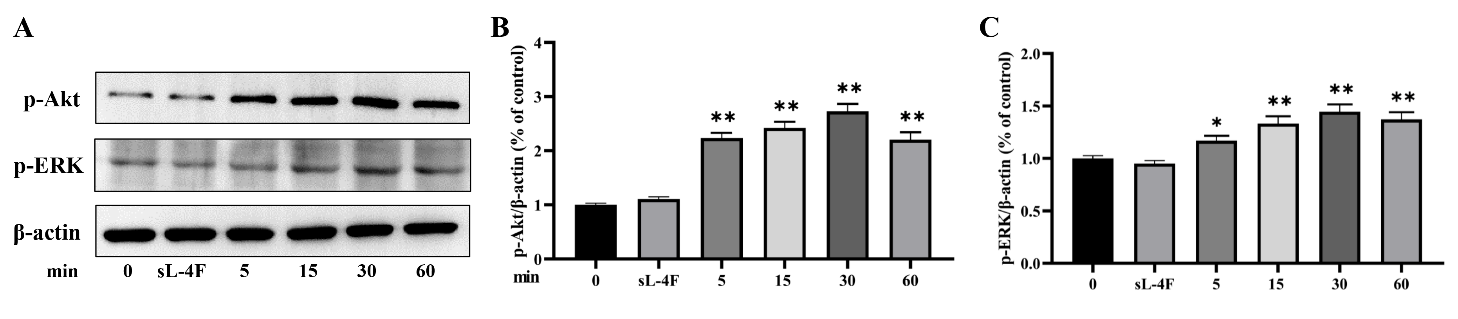
** **Supplementary Figure 3.** Protein levels of p-Akt and p-ERK in HUVECs at different time points. Cells were treated with L-4F (50 μg/mL) for 0, 5, 15, 30, and 60 min, respectively, or sL-4F at 50 μg/mL for 30 min. n=3, one-way ANOVA, Dunnett's test, normalized to β-actin, *P<0.05, **P<0.01, compared with control.

**
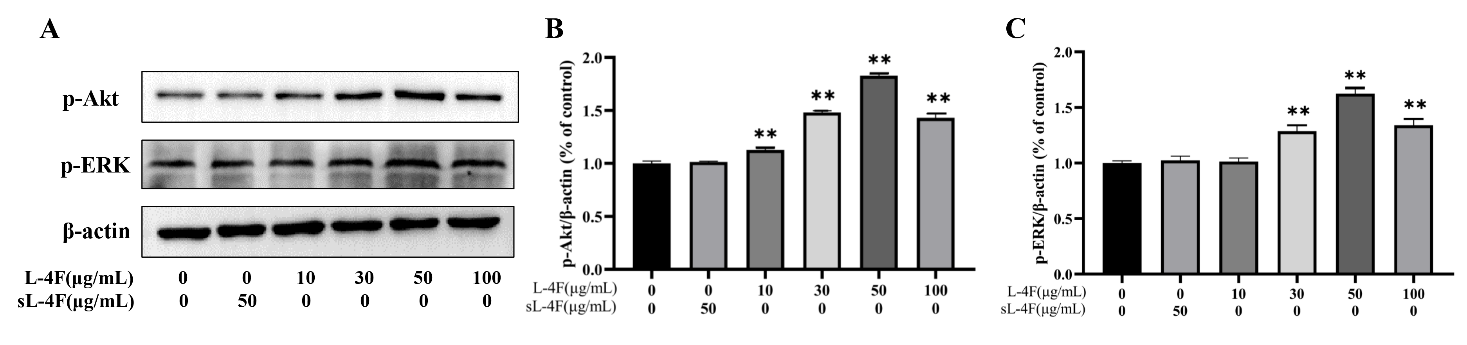
** **Supplementary Figure 4.** Protein levels of p-Akt and p-ERK in HUVECs at different concentrations. Cells were treated with L-4F at 0, 10, 30, 50 and 100 μg/mL, respectively, or sL-4F at 50 μg/mL for 30 min. n=3, one-way ANOVA, Dunnett's test, normalized to β-actin, **P<0.01, compared with control.

**
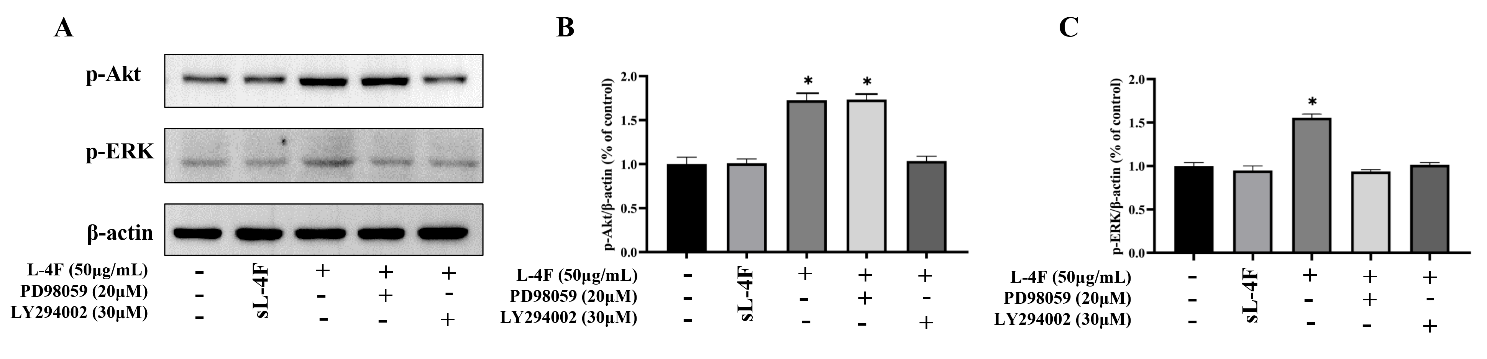
** **Supplementary Figure 5.** Protein levels of p-Akt and p-ERK in HUVECs after pretreatment of LY294002 and PD98059. Cells were pretreated with and without relevant specific inhibitors for 2 h prior to the treatment with L-4F or sL-4F at 50 μg/mL for 30 min. n=3, one-way ANOVA, SNK test, normalized to β-actin, *P<0.05, compared with other groups.
